# To Anticoagulate or Not: Acute Stroke in a COVID-19 Patient With Gastrointestinal Bleed

**DOI:** 10.7759/cureus.10554

**Published:** 2020-09-20

**Authors:** Justin George, Zachary Kirkland, Natalia Lattanzio, Juliette Coleman, David Stone

**Affiliations:** 1 Internal Medicine, Sarasota Memorial Hospital, Sarasota, USA; 2 Internal Medicine, Sarasota Memorial Hospital, Florida State University, Sarasota, USA; 3 Neurology, Sarasota Memorial Hospital, Sarasota, USA

**Keywords:** covid 19, stroke, acute blood loss anemia, complications of anti-coagulation, large vessel thrombosis

## Abstract

Severe acute respiratory syndrome coronavirus 2 (SARS-CoV-2) is a member of the coronavirus family, which comprises enveloped positive sense ribonucleic acid (RNA) viruses responsible for pandemic outbreaks including Severe Acute Respiratory Syndrome Coronavirus (SARS-CoV), Middle Eastern Respiratory Syndrome Coronavirus (MERS-CoV), and most recently coronavirus disease 2019 (COVID-19).

A 30-year-old previously healthy male diagnosed 11 days earlier with COVID-19 presented with right-sided weakness and dysarthria. The patient was found to have an acute left carotid thrombus with embolic multifocal infarcts throughout the left cerebral hemisphere. He was treated acutely with intravenous heparin however developed gastrointestinal bleeding, prompting discontinuation of anticoagulation. Follow up CT angiography 12 days following his stroke demonstrated complete resolution of the thrombus. Since discharge, the patient has been managed with antiplatelet therapy alone with complete neurologic recovery.

Large vessel strokes amongst young patients have been a growing concern during the SARS-CoV-2 outbreak. The use of acute therapeutic and prophylactic anticoagulation is based on risk assessment. Albeit, the utility of anticoagulation in COVID-19 patients remains undetermined.

Prevention of stroke recurrence is a clinical priority for providers treating large vessel stroke patients. More research is required to establish the effectiveness of anticoagulation and antiplatelet therapy for stroke prevention in patients diagnosed with COVID-19.

## Introduction

Severe acute respiratory syndrome coronavirus 2 (SARS-CoV-2) is a virus spread from person to person via close contact. The novel coronavirus disease 2019 is responsible for the illness now designated COVID-19. Since its emergence in China, the virus has rapidly spread throughout the world with current estimates indicating 16 million confirmed cases and approximately 148,000 deaths in the United States [1]. The drastic escalation resulted in the outbreak being declared a pandemic in March 2020.

A myriad of symptoms and pathological processes have been observed amongst those infected. The classic presentation involves pulmonary features such as cough, shortness of breath, and fever, with many patients experiencing flu-like symptoms. While most patients were found to have mild disease, critical illness was reported in 5%. Symptoms of infection have pushed beyond the spectrum of respiratory illness to manifest as cardiac injury, thromboembolic events, and neurologic events [2,3].

Thromboembolic events such as large vessel strokes have been seen at an alarming rate in young patients (<50 years old) who have tested positive for COVID-19. While inflammation and prothrombotic state are associated with the disease, understanding its translation to increased risk of large vessel stroke amongst patients continues to evolve. Current theories include modifications in angiotensin regulation, vascular tropism promoting vasculopathy/vasculitis [4], thrombotic microangiopathy resulting in vascular microdissection [5], and cardioembolic phenomenon from viral-induced cardiomyopathy.

Case series and retrospective studies have highlighted large vessel strokes as a presenting symptom and possible complication secondary to the novel virus [6]. Our unique case examines the course of a young male presenting with ischemic stroke in the setting of SARS-CoV-2 and complicated by a lower GI bleed. Here we discuss the role of anticoagulation in viral-mediated stroke when contraindications such as blood loss anemia exist concurrently.

## Case presentation

A 30-year-old African American male with no reported past medical history initially presented to the emergency department for evaluation of approximately one week of shortness of breath, non-productive cough, along with subjective transient fevers. He initially tested positive for COVID-19 via polymerase chain reaction (PCR) testing from nasopharyngeal swab. He was hemodynamically stable and discharged from the emergency department with instructions to self-quarantine for two weeks.

Eleven days following discharge, the patient developed mild right hemiparesis, sensory loss, and dysarthria. Upon presentation to the hospital, he experienced rapid resolution of his symptoms with National Institutes of Health Stroke Scale (NIHSS) improving from 4 to 1 in two hours. On arrival, the patient was afebrile, with a blood pressure of 143/77 mmHg, pulse of 107 beats per minute, and 18 respirations per minute while saturating 100% on room air. After his symptoms abated, his only complaint was mild left-sided neck swelling he noticed following a burst of coughing spells while self-isolating.

On physical exam, the patient was alert and oriented with equal strength and range of motion bilaterally. Cranial nerves were intact with 2+ deep tendon reflexes throughout. No gait abnormalities, focal neurological deficits, ataxia, or motor/sensory disturbances were observed. Physical exam was remarkable only for minimal left neck swelling with no appreciable cervical lymphadenopathy.

**Figure 1:**
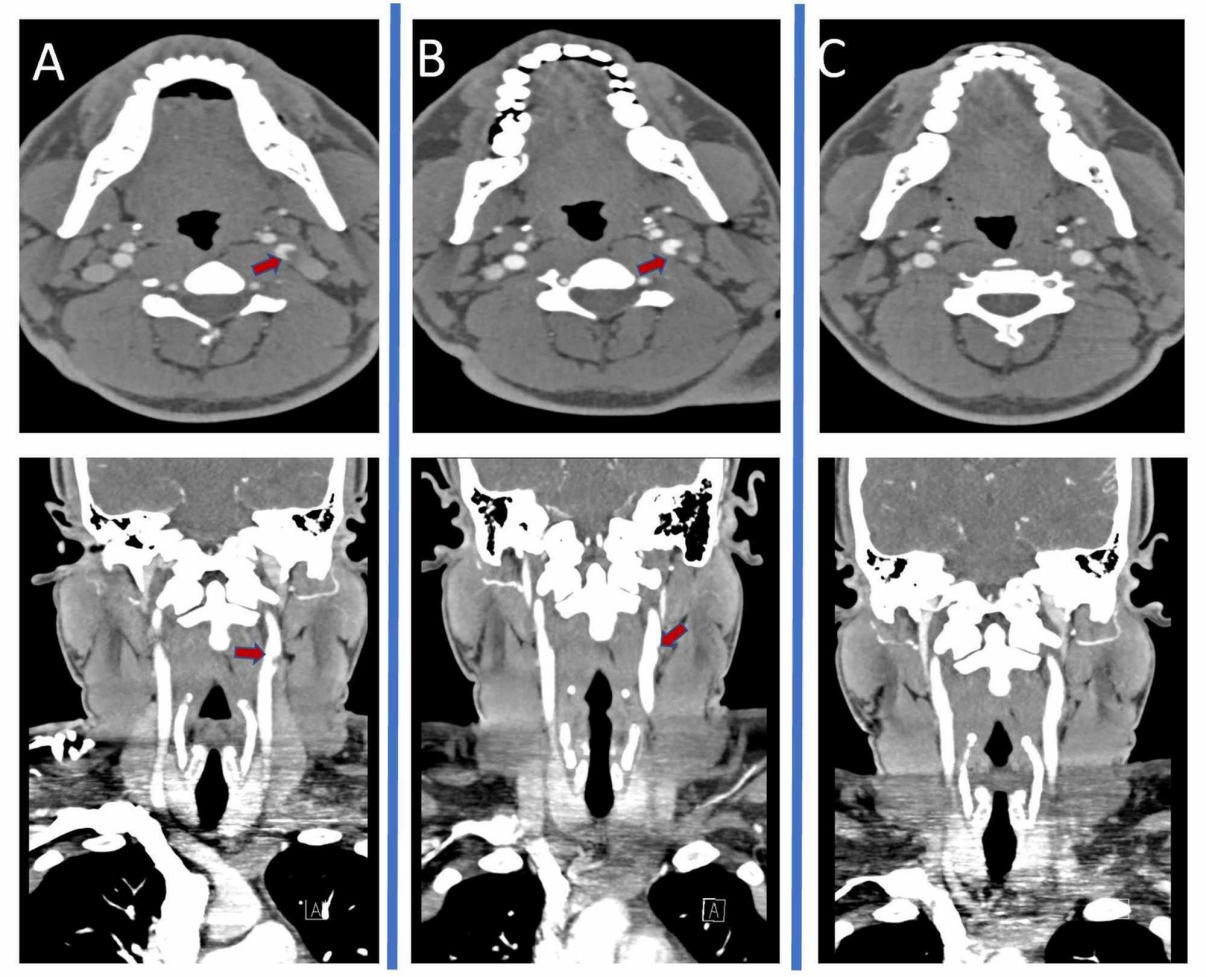
Serial CT angiography (CTA) imaging of Internal Carotid Thrombus A) Axial and coronal CTA on admission  B) Axial and coronal CTA done three days following admission  C) Axial and Coronal CTA done at discharge

**Figure 2:**
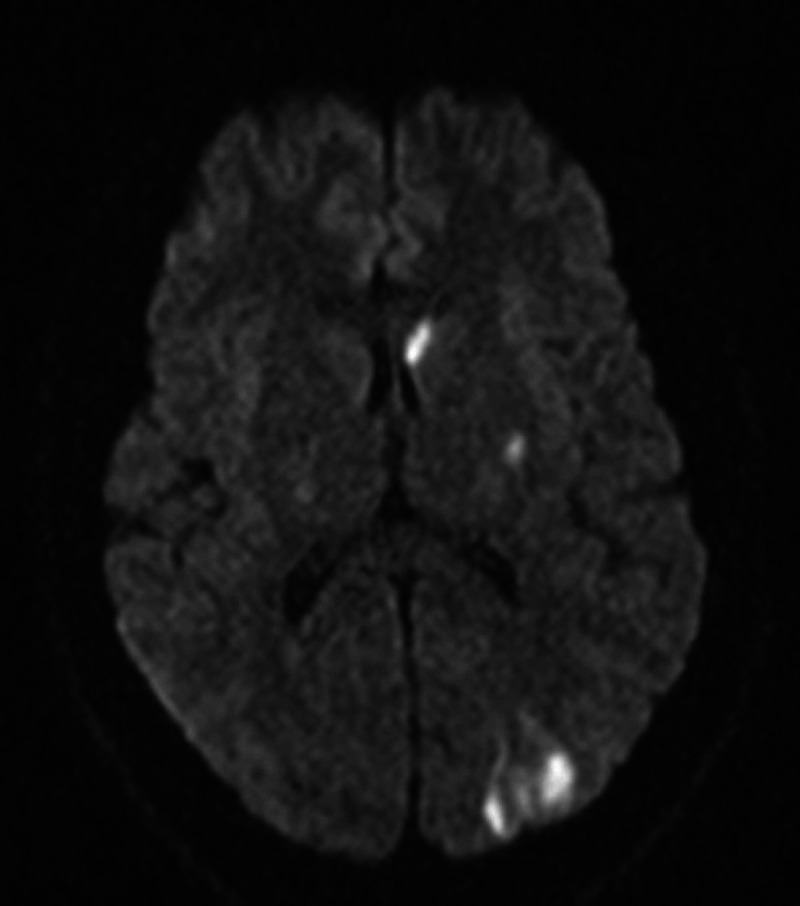
Admission MRI showing multiple hyperintensities suggestive of acute embolic stroke along left middle cerebral artery (MCA) territory

CT head showed chronic appearing hypodensities in the left subinsular cortex, suggesting small chronic infarcts. A nonocclusive thrombus was noted within the proximal left internal carotid artery by CT angiography (CTA) (Figure 1-panel A). Neither CTA nor carotid ultrasonography identified evidence of microdissection. However, multifocal ground glass opacities were visualized within the upper lungs. Chest X-ray however did not identify any acute pulmonary process. MRI brain demonstrated multiple small acute embolic strokes in the left internal carotid artery (ICA) vascular territory (Figure 2). Labs demonstrated a microcytic anemia with a hemoglobin of 8.4 (12.3-15.3 g/dL) and a mean corpuscular volume of 78.4 (80-100 fL), mildly elevated C-reactive protein of 0.4 (<0.3 mg/dL), elevated erythrocyte sedimentation rate at 71 (0-15 mm/hr), and positive COVID-19 via repeated nasopharyngeal swab. D-dimer was elevated at 1.7 (0.0-0.49 mg/L) as was his interleukin-6 at 3.8 (<1.8 pg/mL). Ferritin was abnormally low at 7 (26-388 ng/mL) and iron studies suggested iron deficiency anemia with total iron of 21 (65-175 ug/dL) and percent saturation of 6% (10-55%). 

In light of an intraluminal thrombus, the patient was initially treated with aspirin, statin, and heparin infusion without boluses. However, the patient’s hemoglobin continued to slowly decline over the next three days to 6.9 g/dL, prompting transfusion with two units of packed red blood cells as well as intravenous iron. Upon further questioning, the patient admitted he had noted bright red blood when defecating with intermittent passage of clots confirmed by our healthcare team. He also revealed history of bleeding internal hemorrhoids since age 15 years, not previously reported, and occurring as recently as two to three weeks prior to his stroke. Heparin was discontinued and hemorrhoids were visualized on rectal exam. He was continued on 81 mg of aspirin daily.

Hypercoagulable workup including antinuclear antibodies, factor V Leiden, anticardiolipin, antithrombin III, lupus anticoagulant, protein C and S activity, prothrombin G20210A mutation, and homocysteine were all within normal limits. Transthoracic echocardiography did not identify any intracardiac defects. To clarify his risk of recurrent thrombosis against his bleeding risk, colonoscopy, transesophageal echocardiography, and cerebral catheter angiography were sought. However, despite any respiratory symptoms, our patient repeatedly tested positive for SARS-CoV-2 via PCR on day one, three, and six, deeming the risk of aerosolization too high for such invasive procedures. Antibody testing was pursued to further stratify infectivity which showed the patient had developed immunoglobulin (Ig)G antibodies. Nevertheless, our patient did not have these procedures done inpatient as utility surrounding antibody testing did not comment on infectivity.

Our patient was ultimately discharged in stable condition after multiple studies excluded all other causes of thromboembolic large vessel stroke. Repeat CTA done three days after admission continued to show an intraluminal filling defect (Figure 1-Panel B). Another CTA was obtained twelve days after admission which demonstrated complete resolution of the left carotid thrombus, and without any evidence of new strokes or intracranial hemorrhage (Figure 1-Panel C). At the time of discharge, his hemoglobin had significantly improved following intravenous iron administration and his dimer value had decreased since admission, although remained slightly elevated. He was discharged with daily aspirin and statin for secondary stroke prevention, as well as counseling regarding diet and exercise. At one month after discharge, he had completely recovered without recurrent transient ischemic attack (TIA)/stroke.

## Discussion

Thrombotic complications have continued surfacing in patients with COVID-19. Virchow had stated that thrombosis and thromboembolic disease was attributed to endothelial injury, hypercoagulability, and blood stasis. Preliminary literature has suggested disseminated intravascular coagulation (DIC) as a possible mechanism, as thrombocytopenia and elevated D-dimer has have been found at higher rates in COVID-19 infected patients. Endothelial dysfunction [7], upregulation of procoagulant genes, and Toll-like receptor activation [8] are also hypothesized to occur.

Autopsies performed on patients with COVID-19 demonstrated thrombotic microangiopathy restricted to the lungs with fibrin thrombi visualized within the capillaries and small vessels The high incidence of pulmonary embolus in patients with COVID-19 is suggestive of hypercoagulable state evident in COVID-19 patients. Moreover, similar histologic findings were found in patients infected with SARS-CoV-1 and MERS-CoV. Retrospective studies identified thrombocytopenia as a prominent finding amongst all three members of the coronavirus family [8]. Furthermore, we as well as other centers have noted an increase in the proportion of large vessel strokes during the pandemic.

Defining the mechanism of stroke allows the selection of the right treatment to decrease recurrence. The International Society on Thrombosis and Haemostasis (ISTH) recommended that all hospitalized patients with COVID-19 receive prophylactic low dose molecular weight heparin (LMWH), unless contraindications were present, after improved survival was associated with anticoagulation. Others have argued that the association between anticoagulation and better prognosis was seen in patients meeting severe disease, those with sepsis-induced coagulopathy, or markedly elevated D-dimer [9].

While there was some benefit seen in those with venous thromboembolism, the role of anticoagulation in arterial thrombosis remains less clear. A short course of heparin along with a single antiplatelet agent has been reported to be safe and effective as treatment for a visualized intraluminal thrombus, with the thrombus resolving in the majority of patients in a median of six days [10]. Indeed, this strategy has been used safely and effectively in carefully selected patients found to have an acute intraluminal thrombus with perceived risk for additional embolic events, who are felt to have lower risk of hemorrhagic transformation in our experience as well. Avoiding heparin boluses and changing to an antiplatelet agent(s) alone after imaging demonstrates thrombus resolution reduces long term exposure to risks of anticoagulation. Certain studies have suggested that initial anticoagulation for symptomatic intraluminal carotid artery thrombosis may prevent recurrence [11].

However, full-dose anticoagulation does increase risk of bleeding complications as opposed to antiplatelet therapy alone. Hence, it is not used without careful consideration of hemorrhagic transformation risk or other bleeding complications in acute stroke patients. Hence, it is not recommended for most patients without a visualized intracardiac thrombus, intraluminal thrombus, or other high-risk conditions. National guidelines recommend against the use of full-dose anticoagulation for patients with acute ischemic stroke due to increased bleeding risk and minimal efficacy [12]. As evidenced by resolution of the thrombus on discharge, our patient’s symptoms were secondary to acute intraluminal thrombus opposed to chronic atherosclerotic disease.

Indeed, antiplatelet agents are the preferred treatment initially for acute stroke patients (after any acute reperfusion therapies). The continued resolution of the intraluminal thrombus in this case after anticoagulation was stopped, due to lower GI bleed, suggests that aspirin alone was effective for this patient. We cannot know if the initial treatment with anticoagulation prevented any additional embolic events early in his treatment course. However, the continued resolution of the thrombus does suggest that antiplatelet therapy alone may be sufficient to reduce risk of recurrent stroke, especially in patients at risk for bleeding complications, and even with a visualized intraluminal thrombus felt to be high risk for recurrent stroke.

There are currently no set guidelines for the use of anticoagulation for treatment of large vessel stroke in SARS-CoV-2 positive patients. This is mainly predicated on the unknown mechanism causing prothrombotic states in COVID-19 patients. The present case suggests antiplatelet therapy without anticoagulation may prove effective for COVID-19 patients with stroke with no other identifiable cause. Low hemoglobin in an otherwise healthy individual should prompt additional questioning and examination to rule out recent bleeding before deciding on antithrombotic therapy.

## Conclusions

Anticoagulation utilization in large vessel stroke remains an area of controversy, and antiplatelets are typically the mainstay of treatment in addition to statins and lifestyle modifications. As this novel virus continues to spread, clinicians will be challenged to visit this topic. Our case evinces that in recovering COVID-19 patients with no other identifiable risk factors for thromboembolic stroke, treatment with antiplatelet therapy alone may be an effective choice. In cases with an acute visualized thrombus felt to be higher risk for recurrent stroke, patients should be carefully monitored for any bleeding complications and the choice to anticoagulate should be carefully weighed against risk of bleeding, and in discussion with a vascular neurologist. More data will be needed to establish antiplatelet of choice, length of therapy, and indications for concurrent anticoagulation therapy in this specific population.

## Figures and Tables

**Figure 3:**
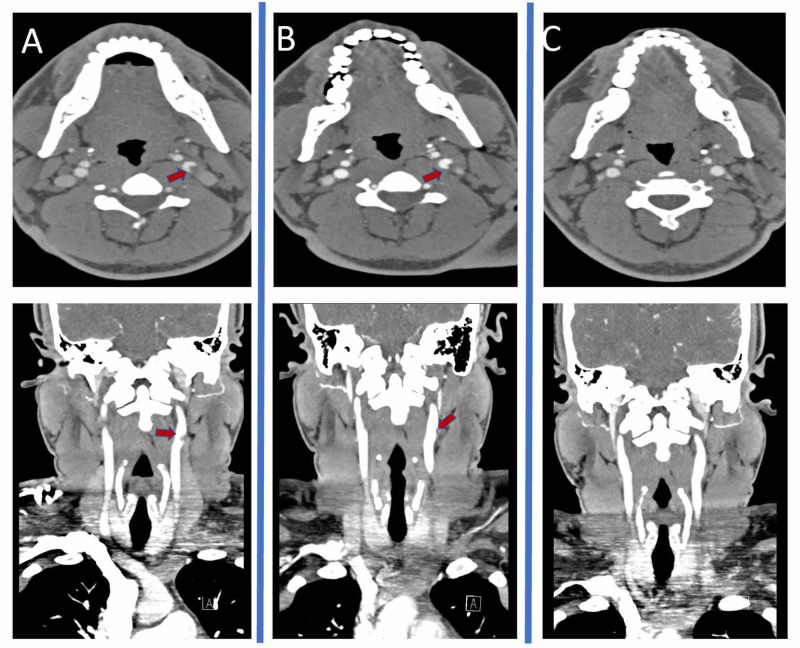
Serial CTA imaging of Internal Carotid Thrombus A) Axial and coronal CTA on admission  B) Axial and coronal CTA done 3 days following admission  C) Axial and Coronal CTA done at discharge

**Figure 4:**
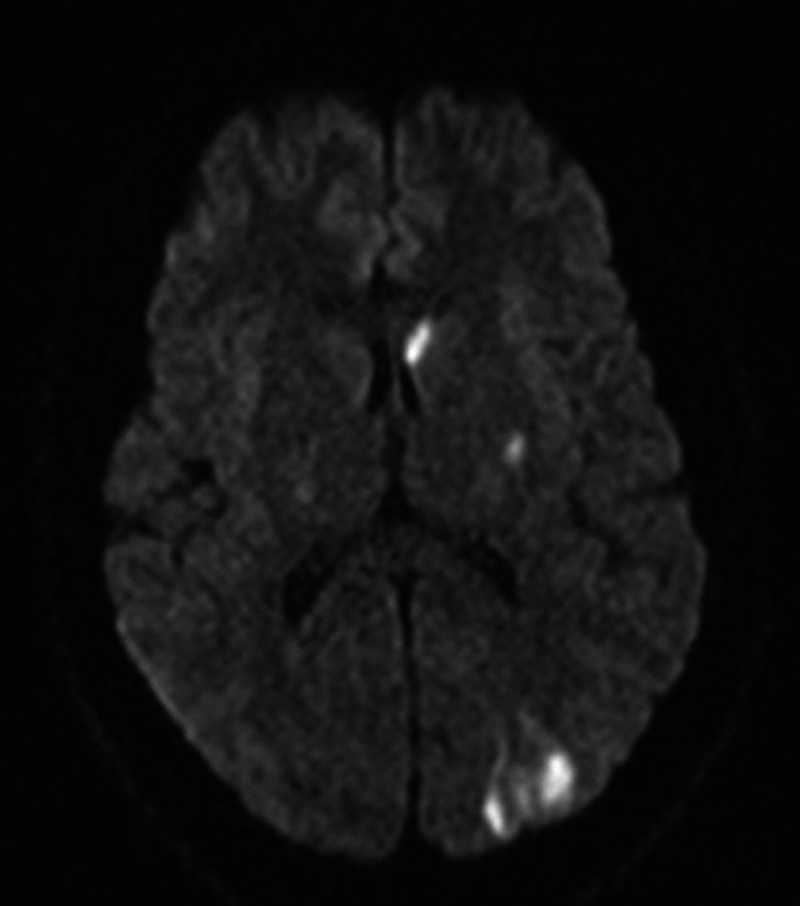
Admission MRI showing multiple hyperintensities suggestive of acute embolic stroke along left middle cerebral artery (MCA) territory.
